# Sorbent Strip Microextraction as a Practical Tool for Drug Screening: Application to Opioids and Local Anesthetics in Human Urine

**DOI:** 10.3390/molecules31040605

**Published:** 2026-02-09

**Authors:** Marisa H. Maria, Thomas Berg, Nuno R. Neng

**Affiliations:** 1 Centro de Química Estrutural, Institute of Molecular Sciences, Departamento de Química e Bioquímica, Faculdade de Ciências, Universidade de Lisboa, 1749-016 Lisboa, Portugal; marisahm1998@gmail.com; 2 Department of Forensic Sciences, Division of Laboratory Medicine, Section of Drug Abuse Research, Oslo University Hospital, 0456 Oslo, Norway; rmthbe@ous-hf.no; 3 Laboratório de Ciências Forenses e Psicológicas Egas Moniz, Egas Moniz Center for Interdisciplinary Research, Egas Moniz School of Health & Science, Quinta da Granja, Caparica, 2829-511 Almada, Portugal

**Keywords:** adsorptive microextraction techniques, HPLC-DAD, opioids, local anesthetics, medication misuse

## Abstract

The present contribution proposes a new design for adsorptive microextraction devices that promote a user-friendly and greener analytical approach. Novel Sorbent Strip Microextraction (SSμE) devices were made using a flexible adhesive film coated with convenient sorbents. Comparing the previous adsorptive microextraction devices, i.e., bar adsorptive microextraction and multi-sphere adsorptive microextraction, the main advantage of the sorbent strip device is its simple design and reduced device preparation time and waste. To demonstrate its applicability, three opioids (buprenorphine, tapentadol, and tramadol) and two local anesthetics (articaine and bupivacaine) were used as model compounds in urine matrices, followed by high-performance liquid chromatography with diode array detector (HPLC-DAD) analysis. Key parameters such as sorbent type, desorption conditions, and microextraction variables were systematically optimized by experimental designs. Under the final conditions, the method achieved recoveries ranging from 78% to 108%, trueness within ±7% and precision expressed by relative standard deviation below 13%. The technique demonstrated good linearity (*r*^2^ ≥ 0.9922) across dynamic ranges of 5–500 ng/mL for local anesthetics and 50–5000 ng/mL for opioids. The validated SSμE/HPLC-DAD methodology was successfully applied to real urine samples, confirming its high precision and accuracy. The proposed microextraction technique offers a practical, eco-friendly, and effective alternative for routine drug screening in complex biological matrices and presents significant advantages over traditional and other microextraction-based methods.

## 1. Introduction

The issue of drug abuse extends beyond the consumption of illicit substances. Psychoactive medications like analgesics, opioid substitution drugs, sedatives and hypnotics are often consumed, potentially resulting in dependency, serious health problems or even death [1,2,3,4]. Medicine misuse is a wide concept that includes various types of harmful consumption, such as increasing dosage of prescribed drugs, concurrent use of products with substances of abuse, and medical accidents and malpractice [5,6,7].

Opioids are medicines used to reduce pain; however, in some cases, their effects may lead to overdose when the dosage taken is above the prescribed amount or in cases of concurrent use with other substances of abuse [8,9]. Local anesthetics are used for pain management during medical procedures, more frequently linked to dental treatments [10]. They are also related to overdose and drug abuse [11]. Their depressant properties in the central nervous system (CNS) make them often used as adulterants of cocaine [12]. Due to being relatively cheap and accessible, they can mimic or increase the effects of cocaine and raise profits by masking the dilution and the product’s poor quality [12,13,14]. Therefore, effective monitoring and regulation of these substances are essential for safeguarding public health. Forensic and clinical laboratories performing routine analyses of biological matrices typically employ techniques such as protein precipitation (PPT), liquid–liquid extraction (LLE), and solid-phase extraction (SPE) [15,16]. While these methods are fast and effective, they often require large amounts of organic solvents and involve high costs and substantial operational effort [17].

In the past few decades, modern analytical chemistry has changed toward greener and more sustainable techniques [18,19]. This includes developing approaches that are miniaturized, simplified, and higher in selectivity and sensitivity, reducing the amount of organic solvent and sample volume [19,20,21]. All these improved attributes align with the principles of green analytical chemistry. Among these advancements, various sorptive-based extraction techniques have been developed and used to extract different classes of organic compounds in different types of sample matrices. Sorptive microextraction is a broad term that encompasses both dissolution and adsorption-based techniques. Common examples include solid phase microextraction, stir bar sorptive extraction, thin-film microextraction (TFME), and bar absorptive microextraction (BAµE), which are frequently used for sample enrichment before chromatographic analysis [22,23,24]. TFME has attracted considerable attention for its efficient extraction performance. In TFME, the extraction phase is confined to a thin film only a few micrometers thick, providing a large surface area to volume ratio that facilitates faster analyte sorbent interactions, leading to shorter equilibrium and extraction times. The thin sorbent film is typically prepared on a solid support using various coating techniques [25,26]. In the case of the BAµE approach, it introduced a static microextraction method based on floating sampling technology. This method uses nanostructured materials, which are very effective for trace analysis of medium-polar to polar compounds in aqueous media [22]. Another advantage is the possibility of the selection of sorbent phases, which can be activated carbons or polymers, allowing for adaptability for specific compounds [27,28,29,30].

Building on the principles of BAμE and TFME, this study proposed and validated a more simplified and user-friendly technique: Sorbent Strip Microextraction (SSμE). This new approach employs simple microextraction strips coated with an appropriate sorbent phase of choice suited to the compounds of interest. SSμE uses commercial sorbent phases and adhesive tape while improving simplicity and scalability. The design allows easy incorporation of various sorbents through “sticking-based technologies”, making it adaptable for a wide range of target compounds. The main objective of the present study was to develop and apply the SSμE technique for the determination of three opioids (buprenorphine, tapentadol, and tramadol) and two local anesthetics (articaine and bupivacaine) in human urine. In addition, we aimed to evaluate whether the technique qualifies as an effective, green, rapid, and reliable analytical extraction approach.

## 2. Results and Discussion

### 2.1. SSµE Devices

The SSμE technique represents a third generation of adsorptive microextraction that is specifically distinguished from previous generations, such as BAμE and multi-sphere adsorptive microextraction (MSAµE), by its structural shift from rigid supports to a flexible design. While earlier methods relied on rigid substrates like polypropylene tubes or polystyrene spheres, SSμE utilizes a flexible adhesive film coated with powdered sorbents. This “sticking-based” approach allows the devices to be easily “lab-made” through a simple manual coating process, significantly reducing preparation time.

**Figure 1:**
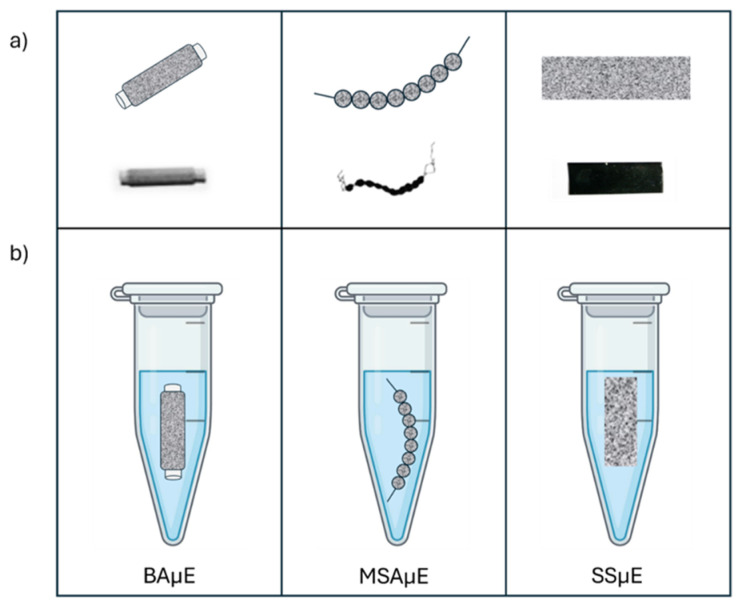
Schematic representation and photographic images of the BAµE, MSAµE, and SSµE devices proposed in the present study (**a**) and illustration of their operation during the microextraction process (**b**) [31].

As described in the Experimental Set-Up, SSµE followed the same principles of BAµE using adhesive films to fix the sorbent phases. Figure 1 depicts schematic representations of BAµE, MSAµE and SSµE devices with sorbent phases, as well as their use during the extraction process in Eppendorf tubes for a thermoshaker. Stability tests were performed to evaluate the behavior of the SSµE adhesive supporting films during the extraction and back extraction processes compared to BAµE. As expected, the assessment showed, in general, good stability for both devices during the extraction with the thermoshaker. Additionally, it was found that SSµE uses the same amount of sorbent as BAµE but requires significantly less adhesive film, which minimizes waste. This indicates that a single band of adhesive film can give more for SSµE devices than BAµE, offering greater material efficiency. For back extraction, the interaction with organic solvents such as methanol (MeOH) and acetonitrile (ACN) was tested, in which no occurrence of desegregation of the sorbent phases was observed.

### 2.2. HPLC-DAD Optimization

The first step of this study was to establish instrumental conditions more appropriate for analyzing the local anesthetics and opioids. The HPLC conditions optimized were gradient profile, column temperature and injection volume. For DAD and according to the UV/vis data, the wavelengths (λ_max_) with maximum response for articaine and the remaining four compounds were 280 nm and 210 nm, respectively. These optimizations allowed for efficient resolution and good responses for all five analytes, with a total analysis time of 24 min.

The calibration was performed by injecting standard solutions (7 levels) having concentration from 0.05 to 5.0 µg/mL (for articaine and bupivacaine) and 0.5 to 50.0 µg/mL (for tramadol, tapentadol and buprenorphine). The calibration curves’ determination coefficients (*r*^2^) were ≥0.997 for all the compounds. The instrumental LODs were 0.1 µg/mL for bupivacaine and articaine and 2 µg/mL for buprenorphine, tramadol and tapentadol. For the LOQs the values were 1.0 µg/mL (bupivacaine and articaine) and 10µg/L (tramadol, tapentadol and buprenorphine). The instrumental precision was evaluated by consecutively injecting a standard mixture (*n* = 6, 3–30 µg/mL), resulting in relative standard deviations (RSDs (%)) lower than 5.7%. In addition, no carryover was observed in the blank runs, for which the background observed was consistently below the LODs.

### 2.3. Optimization Assays

#### 2.3.1. Sorbent Phase Selection

**Figure 2:**
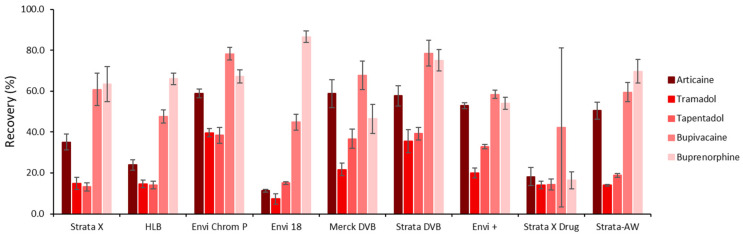
Average recovery yields obtained for the five target compounds using different polymeric phases in aqueous media. The error bars represent the standard deviation of three replicates.

To select the most suitable sorbent phase for SSµE, nine polymeric phases were tested (HLB, Envi+, Envi 18, Envi ChromP, Merck-DVB, Strata-DVB, Strata-X, Strata-X Drug and Strata-AW) under the standard experimental conditions chosen. Figure 2 shows the recovery obtained for each polymer. Among all the sorbents tested, both DVB-based polymers and Envi ChromP gave the highest average recovery for the analytes. Strata-DVB was selected for further experiments due to its slightly better recovery yields.

The Strata-DVB phase is a styrene-divinylbenzene polymer that remains stable across a pH range of 1–14. It enhances the retention of analytes based on particle size and surface area, with the surface structure favoring hydrophobic and aromatic interactions, resulting in strong retention of non-polar or moderately polar analytes. The compounds in this study are from two different classes of drugs with different physicochemical properties. The local anesthetics have both a hydrophilic amino group (tertiary or secondary) and a lipophilic aromatic ring. The opioids studied include semi-synthetic buprenorphine (a derivate of morphine) and the synthetic tramadol and tapentadol. They are all polar compounds with different polarities. Tramadol (log *p* 1.34–2.4) and tapentadol (log *p* 2.96–3.47) are moderately polar, and buprenorphine has a higher lipophilicity (log *p* 3.8–4.98).

Local anesthetics, while partly hydrophilic, contain a lipophilic aromatic ring that interacts well with the sorbent, particularly through aromatic and hydrophobic mechanisms. Similarly, buprenorphine is highly lipophilic, with strong hydrophobic binding, which also shows good retention. These interactions led to high recoveries, above 50%. Contrarily, tramadol and tapentadol are both hydrophilic and moderately polar. As a result, they exhibit a stronger affinity for aqueous environments, leading to weaker interactions with the hydrophobic/aromatic sorbent, which resulted in recoveries around 30%. This difference can be attributed to their molecular structures, which include more polar functional groups, such as hydroxyl and amine groups. Therefore, to achieve higher recoveries for all the compounds, especially for these last two compounds, it was necessary to optimize parameters such as pH, ionic strength and others.

#### 2.3.2. Back Extraction

**Figure 3:**
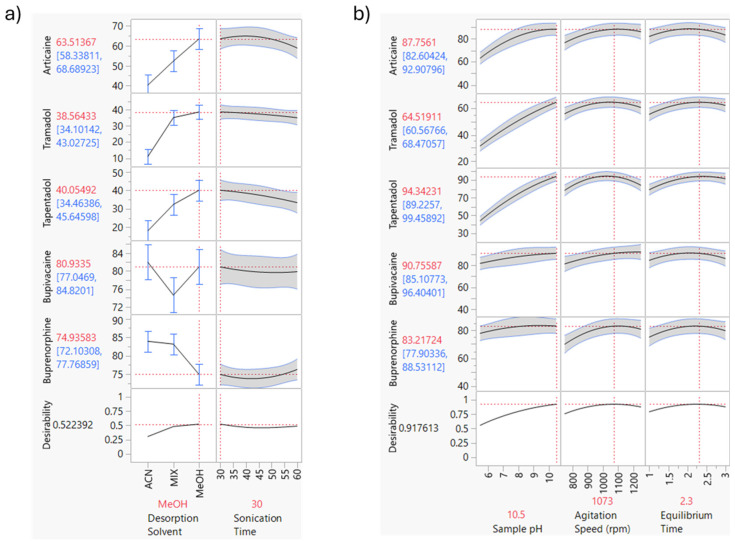
(**a**) Full factorial design of the back extraction stage tested MeOH with a desorption time of 30 min and (**b**) Box–Behnken factorial design for testing of three extraction parameters: sample pH (pH 5.5; pH 8.0; pH 10.5), agitation speed (750; 1000; 1250 rpm) and equilibrium time (1 h; 2 h; 3 h). Solid black curves represent the model-predicted mean response based on the fitted DOE model. Blue solid curves indicate the 95% confidence interval for the mean prediction. Gray shaded bands represent the wider 95% prediction interval, reflecting experimental variability. Red dashed vertical lines mark the selected/optimal factor settings, and red dashed horizontal lines indicate the corresponding predicted response values at those settings. Blue error bars on categorical factors reflect observed experimental means ± variability. The *Y*-axis for each compound shows the percent recovery. For desirability, it is represented on a scale from 0 to 1, where 1 indicates the most desirable outcome and 0 indicates the least desirable.

The compound’s recovery in the back extraction stage is influenced by factors like liquid desorption solvent and sonication time. The liquid desorption solvent must have enough strength and allow sufficient time to fully desorb the analytes from the sorbent, a process that can be enhanced by sonication. To investigate the back extraction step, three different liquid desorption solvents, MeOH, ACN, and ACN/MeOH (1:1, *v*:*v*), and desorption times were tested using a full factorial experimental design. As shown in Figure 3a, the prediction profiler using MeOH as the desorption solvent with 30 min desorption time yielded recoveries between 38% and 80%.

#### 2.3.3. SSµE Parameters

Several parameters affect the SSµE efficiency, such as equilibrium time and agitation speed, but the chemical characteristics of the matrix also need optimization, in particular, the pH, ionic strength and polarity. Through a Box–Behnken design the extraction velocity, matrix pH, and equilibrium time were tested, with three levels each. Figure 3b shows the conditions that gave the best extraction efficiency: a matrix pH of 10.5 and an extraction time of 2 h and 18 min at 1073 rpm. Under these conditions, recoveries were between 64% and 94%.

The pH optimization was conducted using three discrete levels of pH, 5.5, 8.0, and 10.5, as required by the Box–Behnken design to characterize the experimental space and identify the mathematical optimum. This range was specifically chosen based on the physicochemical properties (p*K*_a_) of the target compounds and the possible degradation of the adhesive film from the microextraction device. At higher pH, increases in the recoveries of articaine, tramadol and tapentadol were observed. These compounds had basic functional groups with p*K*_a_ values of 7.8 [32], 9.4 and 9.3, respectively, and at pH 10.5 they were less ionized and therefore stronger bonded to the non-polar sorbent and less soluble in the aqueous sample. The distribution chart showing the concentration of different molecular species (microspecies or macrospecies) as a function of pH between 0 and 14 is presented in Supplementary Figure S1. As can be observed, at pH values above 10.5, most target analytes are in their anionic form, which is likely to reduce extraction efficiency.

Next, the ionic strength and polarity of the compounds were tested. The addition of salt (NaCl) to a solution increases the ionic strength. This increased ionic strength competes for water molecules, consequently minimizing the solubility of the water with other polar or charged molecules. As a result, the compounds migrate from the matrix to the sorbent, a phenomenon known as the “salting-out” effect [30,33,34]. Additionally, adding an organic modifier (MeOH) to the sample helps prevent the non-polar analytes from adsorbing to the vials [30,33,34]. The results obtained by testing the influence of the addition of NaCl and MeOH in the sample are in Figure S2. From the data obtained, all the compounds benefited from the addition of salt, particularly tramadol and tapentadol, whose recoveries increased more than 20%. Based on this test, a concentration of 10% NaCl was chosen. The addition of an organic modifier also improved the recoveries, although it also led to higher RSD values. Therefore, 5% MeOH was chosen, providing recoveries between 63% and 97% with an RSD below 13%.

After optimization of all the parameters, the method was applied to the selected biological matrix, urine, and compared against ultrapure water. In Table 1, the results demonstrate that the recoveries in urine remained consistent, ranging from 78% to 108% with RSDs below 5%. A slight interference was observed in the retention time of tramadol. However, by using the matrix of choice, the overall extraction efficiency was maintained. This stability indicates that the SSμE technique effectively mitigates significant matrix effects, providing a reliable alternative for trace analysis in biological fluids.

#### 2.3.4. Validation of the Proposed Methodology

The proposed SSμE/HPLC-DAD methodology was validated using blank urine samples to ensure its reliability for routine screening. The selectivity of the method was evaluated by comparing the chromatograms of blank urine, spiked urine, and real clinical samples from volunteers. The analysis confirmed that there were no endogenous interfering substances at the specific retention times of the five target analytes. The use of optimized HPLC-DAD wavelengths at 210 nm and 280 nm provided efficient resolution for both opioids and local anesthetics, further ensuring analytical specificity.

The signal-to-noise (S/N) ratio was used to determine the limit of detection (LOD) and limit of quantification (LOQ) values, where the S/N for LOD had to be ≥3 and the S/N for LOQ had to be ≥10. The LOD values were between 50 and 500 ng/mL, and the LOQs were between 150 and 1500 ng/mL for local anesthetics and opioids, respectively. The linearity range was assessed between 50 and 500 ng/mL for the local anesthetics and 500 and 5000 ng/mL for the opioids, using seven concentration levels analyzed in triplicate. The calibration curves showed good linearity, with *r*^2^ ≥ 0.9922.

In addition, intra- and inter-day trueness and precision were determined at three spiking levels and are presented in Table S1. The precision for the intra-day assays (repeatability) was between 1.6% and 5.2%, and for inter-day assays (intermediate precision during a week) was between 2.2% and 12.7%. The intra-day trueness was below ±3.6%, and the inter-day trueness was lower than ±5.7%.

Although the current validation demonstrates high selectivity and sensibility using DAD, the future integration of SSμE with mass spectrometry could further enhance analytical selectivity and confirmatory power while potentially reducing the required sample volume even further.

#### 2.3.5. Real Samples

Urine samples from six anonymous donors were analyzed by the developed method. A chromatogram of a spiked urine sample containing all the target compounds at the highest concentration level, a chromatogram of a positive sample with articaine, and a blank urine sample are shown in Figure S3. These chromatograms show that the method achieves good selectivity, with no interfering substances at the retention times of the analytes of interest. The results from the analysis of the urine samples are presented in Table 2. These results also indicate that articaine is found in urine at higher concentrations several hours after a dental appointment, especially when compared to its blood half-life of approximately 20 min. Consequently, clearance time is also influenced by the type of injection administered and the presence of metabolites; for example, articainic acid exhibits a half-life of 2 to 2.5 h in urine. The total recovery of the administered dose in urine ranges from 50% to 92% over 36 h [35].

#### 2.3.6. Greenness Assessment

**Figure 4:**
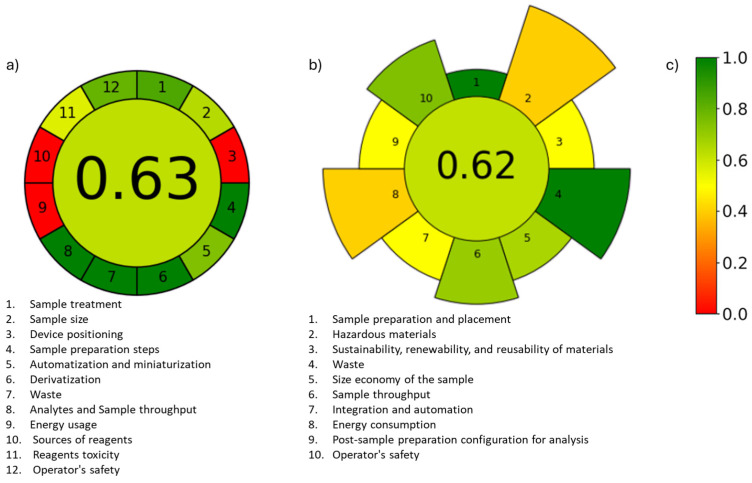
Evaluation of the method and sample preparation using the AGREE calculator (**a**), the AGREEprep calculator (**b**), and the corresponding color scale for reference (**c**).

Analytical Greenness Metric Approach and Software (AGREE) addresses the instrumental and reagent components of analytical methods [36]. In contrast, Analytical greenness metric for sample preparation (AGREEprep) provides a comprehensive evaluation of the environmental impact of sample preparation procedures [37]. The overall score is displayed at the center of the pictogram, where values close to 1 and a dark green color indicate a greener procedure (Figure 4). Based on the AGREEprep assessment and AGREE, the proposed method achieved overall scores of 0.62 and 0.63, respectively. These scores indicate an alignment with green chemistry trends, while some steps require improvement. Specifically, the main limitations of the overall method are energy consumption and the hazards associated with reagent sources.

#### 2.3.7. Comparison with Other Methodologies

Prior to analysis it is usual to conduct a pretreatment of the sample in order to extract the analytes of interest. Some common techniques used are LLE and SPE. However, these approaches do not comply fully with green analytical chemistry principles, even though nowadays they have diminished the amounts of organic solvent used. Table 3 presents several extraction techniques that include the analytes of our study. Although some achieved comparable extraction times to our method, they often require skilled operators or several steps. For instance, methods involving liquid phase microextraction with back extraction (LPME–BE), binary solvents dispersive liquid–liquid microextraction (BS-DLLME), dispersive micro-solid phase extraction (DMSPE) or hydrolysis followed by supported liquid extraction (SLE) are labor-intensive and demand expert handling [38,39,40,41,42,43,44,45]. In comparison, SSµE uses only 100 µL of solvent, reinforcing its alignment with green analytical chemistry principles. In addition, its miniaturized format and low solvent consumption make it an environmentally sustainable option, besides being operator-friendly and requiring minimal technical expertise.

Therefore, when observing the results achieved by the SSμE/HPLC method, it shows high potential as an alternative method for forensic or clinical toxicological analysis. Notably, by integrating SSμE with modern analytical instrumental systems, even better performance can be achieved, namely, in sensitivity and analytical selectivity, with the potential to reduce the sample volume even more.

## 3. Materials and Methods

### 3.1. Chemicals, Standards and Materials

MeOH LC-MS grade (MeOH, purity 99.99%) was purchased from Fisher (Bishop Meadow, UK) and acetonitrile LC-MS grade (ACN, purity 99.99%) from Supelco (Darmstadt, Germany). Sodium chloride (NaCl, purity 99.5%), sodium carbonate (Na_2_CO_3_, purity 99.5%) and sodium bicarbonate (NaHCO_3_, purity 99.7%) were obtained from Sigma-Aldrich (Darmstadt, Germany). Hydrochloric acid was purchased from Merck (Darmstadt, Germany). Formic acid (99%) was acquired from CARLO ERBA Reagents (Chaussée du Vexin, France). Sodium hydroxide (NaOH, 98.0%) was from Sigma-Aldrich (Darmstadt, Germany). Articaine chloride and bupivacaine HCl were purchased from Sigma-Aldrich (Darmstadt, Germany).

Standard solutions were prepared using three pharmaceutical tablets available in the Portuguese market. Tramal 50 mg capsules produced by Grünenthal, S.A. (Algés, Portugal) contained 50 mg Tramadol hydrochloride; PALEXIA produced by Grünenthal, S.A. (Algés, Portugal) contained 50 mg of Tapentadol; and Buprenorfina Azevedos produced by Azevedos (Alcabideche, Portugal) contained 8 mg of Buprenorphine. These stock solutions were prepared according to previously described protocols [46]. Each tablet was individually meshed and dissolved in MeOH, giving final concentrations of 800 mg/L (buprenorphine) and 5000 mg/L (tramadol and tapentadol). Then, the solutions were sonicated (42 ± 2.5 kHz, 100 W, Branson 3510E-DTH, Danbury, CT, USA) for 15 min, centrifuged at 3000 rpm for 10 min and filtered (0.45 μm nylon filters, Labfil, Hangzhou, Zhejiang PR, China). Individual stock solutions were stored at −20 °C in amber glass flasks. Regarding articaine and bupivacaine, the stock solutions were prepared at 100 mg/L by proper dilution with MeOH and stored at 4 °C in amber glass flasks.

The buffer solution was prepared by dissolving the Na_2_CO_3_-NaHCO_3_ in distilled water, adjusting with HCl or NaOH when necessary. The respective pH values were measured with a pH meter (MeterLab^®^ PHM210 Standard pH Meter, Terni, Italy). The sorbent phases used for coating the SSµE devices were Strata SDB-L (SDVB) (reversed phase styrene-divinylbenzene polymer; particle size 100 μm, 260 Å pore size, 500 m^2^/g surface area); Strata-X (Polymeric Reversed Phase N-Vinylpyrrolidone, particle size 33 µm); Strata-X-AW (Polymeric Weak Anion, particle size 33 µm); and Strata-X-Drug B (Polymeric Strong Cation, particle size 33 µm) from Phenomenex (Torrance, CA, USA). Oasis HLB (reversed phase copolymer polypropylene; particle size 30 μm, 80 Å pore size) was from Oasis (Lisbon, Portugal). Supelclean™ ENVI™-18 SPE (PE frit (20 μm porosity) polypropylene, particle size 45 μm, 60 Å pore size) and Supelclean™ ENVI™-Chrom P (polystyrene-divinylbenzene phase, particle size 80–160 μm, 20–300 Å pore size) were from Supelco (Park Bellefonte, PA, USA). ISOLUTE^®^ ENV+ (Hydroxylated Polystyrene-divinyl Benzene Copolymer, particle size 90 μm, 800 Å pore size) from Biotage (Hengoed, UK). Merck DVB (Poly(styrene-co-divinylbenzene), particle size 6.0–10.0 μm, 60 Å pore size), was from Merck KgaA (Darmstadt, Germany).

### 3.2. Urine

Two urine samples were obtained from volunteers who had undergone anesthesia during dental care and collected after 2 h and 4 h after administration. Four additional unknown urine samples were obtained without knowledge of prior substance consumption. All the samples were provided anonymously, with no personal information about the donors. For method validation, blank urine samples were provided by volunteers who guaranteed not to have consumed any of the substances under study. Upon arrival at the laboratory, all the samples were stored at −80 °C until use. Before use, the biological samples were filtered with a 0.45 µm (PTFE) syringe filter (Labfil, Taizhou, China). No additional pretreatment was required.

### 3.3. Instrumental Set-Up

HPLC-DAD analysis was carried out on an HPLC-DAD (Agilent 1260 Infinity II Series LC system, Waldbronn, Germany), which consisted of the following modules: 1260 quaternary pump (G7111B), 1260 vial sampler (G7129A), and the 1260 diode array detector (G7115A). The data acquisition and instrumental control were performed using the OpenLab CDS software (version 2.6, Agilent Technologies, Waldbronn, Germany). Analyses were performed using an Inertsil ODS-3 HPLC Column (150 × 4.6 mm; 5 μm; GL Sciences Inc., Tokyo, Japan) at a temperature of 30 °C. The mobile phase consisted of acetonitrile (solvent A) and 0.1% formic acid aqueous solution (solvent B) with the following gradient: 0–1 min: 5% A; 01–7 min: 5–30% A; 7–12 min: 30–50% A; 12–16 min: 50% A; 16–21 min: 50–5% A; and 21–24 min: 5% A, using a flow rate of 1.0 mL/min. The injection volume was 10 μL at a draw speed of 200 μL/min. The detector was set at 210 nm and 280 nm.

### 3.4. Experimental Set-Up

#### Preparation of the SSµE and Optimization Assays

**Figure 5:**
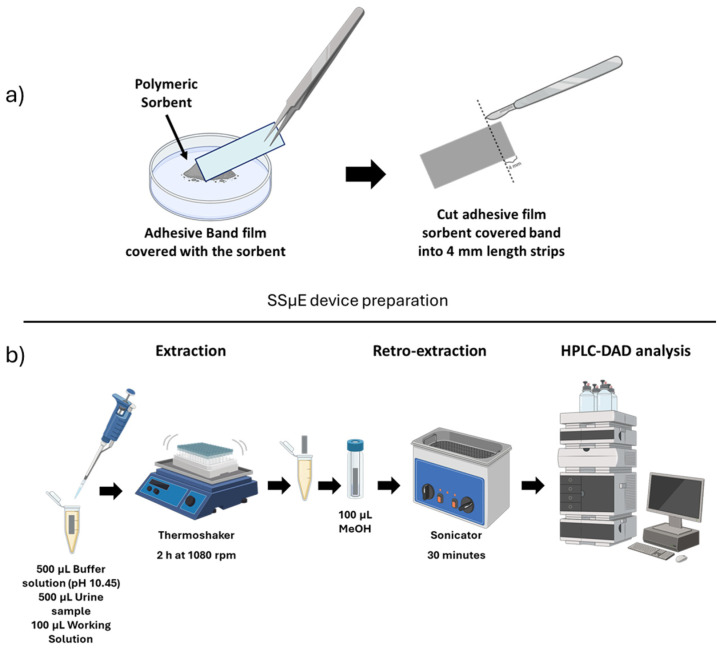
Preparation of SSµE device (**a**) and schematic drawing of sample preparation using SSµE (**b**) [31].

The SSµE devices were lab-made at the moment of use using an adhesive film band, followed by covering it with the powdered sorbents inside a container through manual coating (Figure 5a). Each band was cut into small strips of 4 mm length. The strips went through a clean-up process before use, involving ultra-pure water and MeOH.

Different parameters for SSµE were optimized to achieve optimal conditions for the extraction of the opioids and anesthetic drugs. First, nine different polymeric sorbent materials available in the laboratory were tested. The assay conditions were as follows: in a 2 mL Eppendorf tube, 1 mL of Type 1 water was added and then spiked with 100 µL of a working solution with all the compounds (Figure 5b). An SSµE device of 4 mm was then introduced into the tube, and the extraction was carried out for 1 h at 1000 rpm using a thermoshaker (Biosan, Riga, Latvia) at room temperature. Following extraction, the device was removed and placed into a vial with an insert, to which 100 µL of MeOH was added. Back extraction was performed using ultrasonic treatment (TPC Advance Dentsonic Ultrasonic Cleaner, City of Industry, CA, USA) for 30 min. After this process, the vials were placed in the HPLC-DAD instrument for analysis. After selecting the optimal sorbent material for the extraction of the compounds under study, additional parameters were systematically studied using experimental design approaches and evaluated with JMP Statistical Discovery LLC software (Student Edition 19, Cary, NC, USA).

Initially, the back-extraction stage was optimized using a full factorial design. Three independent factors were selected: liquid desorption mode (X1: ultrasonication time), extraction solvent (X2: MeOH, ACN or ACN/MeOH (1:1, *v*:*v*)), and liquid desorption time (X3: 30 min, 45 min or 60 min). The SSµE was optimized using a Box–Behnken design for the following three factors selected as independent variables: extraction velocity (X1: 750 rpm, 1000 rpm, 1250 rpm), matrix pH (X2: 5.5, 8, 10.5), and equilibrium time (X3: 1 h, 2 h, 3 h). In the last optimization steps, the influence of matrix ionic strength (5%, 10% and 15% NaCl) and the addition of an organic modifier (5%, 10% and 15% MeOH) were assessed. All the experiments were performed in triplicate. Figure 5b presents the experimental scheme of the proposed methodologies.

### 3.5. Validation Assays

Method validation included selectivity, analytical thresholds (LOD and LOQ), linearity, trueness, precision and matrix effects. All the assays were performed under optimized conditions and in triplicate. Each assay involved adding 100 µL of working solution to a mixture consisting of 10% NaCl and 5% MeOH, with 0.5 mL of blank urine and 0.5 mL of 0.5 M Na_2_CO_3_·NaHCO_3_ buffer solution. Calibration standards were prepared at concentrations between 5 and 500 ng/mL for articaine and bupivacaine, and between 50 and 5000 ng/mL for tramadol, tapentadol and buprenorphine to assess the linearity and the coefficients of determination (*r*^2^). Intra- and inter-day trueness and precision were assessed on the same day with six replicates, and over three consecutive days using three concentrations levels, 15, 25 and 45 ng/mL (articaine and bupivacaine) and 150, 250, and 450 ng/mL (tramadol, tapentadol and buprenorphine), in urine.

## 4. Conclusions

In this study, a novel analytical approach, Sorbent Strip Microextraction (SSμE), was developed, optimized, and successfully applied for the determination of three opioids (buprenorphine, tramadol, and tapentadol) and two local anesthetics (articaine and bupivacaine) in human urine. Based on the principles of BAμE and TFME, the SSμE technique offers a simplified, eco-friendly, and cost-effective alternative for trace analysis in biological matrices.

The SSμE method demonstrated several advantages, including customizable sorbent phases, ease of device preparation, cost-effectiveness, low solvent consumption, and compatibility with various analytical techniques. Optimization of sorbent material, extraction conditions, pH, ionic strength, and organic modifiers for SSµE was performed by experimental design. Under the optimized conditions, recoveries ranged from 78% to 108%. Additionally, trueness (≤±7%) and precision (≤13%) were achieved. The validation results confirm the method’s strong analytical performance, with high sensitivity and precision across all the target compounds. In addition, the method demonstrated good linearity and a good determination coefficient (*r*^2^ ≥ 0.9922), as well as excellent selectivity and accuracy.

Application to real urine samples from six volunteers further confirmed the method’s suitability for application in analytical laboratories. The SSμE approach proved effective in identifying and quantifying the target drugs, with minimal matrix interference. Furthermore, when compared to other sorptive-based or liquid-phase microextraction approaches, such as SPE, LPME-BE, or DMSPE, SSμE is notably more operator-friendly, requiring minimal technical expertise and fewer labor-intensive steps. From an environmental standpoint, it aligns more closely with Green Analytical Chemistry principles by using only 100 µL of solvent for desorption, providing a more sustainable and cost-effective alternative for routine screening of psychoactive substances in complex biological matrices.

Overall, SSμE represents a significant advancement in microextraction techniques, combining the benefits of sorptive-based methodologies with practical improvements in simplicity, sustainability, and analytical reliability.

## Figures and Tables

**Table 1:** Recovery yields and %RSD values obtained for each compound in aqueous and in urine matrices.

	Water		Urine	
Compounds	Recov. (%)	RSD%	Recov. (%)	RSD%
Articaine	88	2	88	2
Tramadol	77	4	108	4
Tapentadol	93	2	83	1
Bupivacaine	89	3	78	5
Buprenorphine	75	7	85	2

**Table 2:** Calculated concentrations from 7 urine samples using SSµE/HPLC-DAD methodology.

Sample	Analytes Identified	Compound Amount (µg/mL)
1 ^[a]^	Articaine	7.8
1 ^[b]^	Articaine	33.8
2 ^[a]^	Articaine	18.4
2 ^[b]^	Articaine	26.9
3	Articaine	No compounds identified
4	Articaine	No compounds identified
5	Articaine	No compounds identified
6	Articaine	4.4

^[a]^ Sample taken from a volunteer 2 h after a dentist appointment and ^[b]^ sample taken from a volunteer 4 h after a dentist appointment.

**Table 3:** Comparison of the proposed methodology with other extraction techniques for the determination of local anesthetics and opioids in urine samples.

Analytes	Sample Amount Urine	Solvent Usage	Work Time	Sample Technique	Detection Limits	Recov.	Analytical Method	Ref.
Articaine Bupivacaine	500 µL	100 µL	3 h	SSµE	0.005–0.5 µg/mL	78–108%	HPLC-DAD	This work
Buprenorphine Tapentadol Tramadol					0.05–5 µg/mL			
Buprenorphine	100 µL	-	5 min	Dilute + Shoot	0.005–1 µg/mL	-	LC-MS/MS	[42]

Articaine	1 mL	-	5 min	Dilute + Shoot	0.32–8.34 µg/mL	98%	Electrochemical Determination	[43]
Tramadol	-	2.1 mL	40 min to 1 h	LPME–BE	0.0003–0.13 µg/mL	64%	LC-MS/MS	[38]
Tramadol	5 mL	drops	30–45 min	BS-DLLME	0.001–0.13 µg/mL	95.6–99.6%	HPLC-FLD	[39]
Buprenorphine	5 mL	50 µL	30–45 min	DMSPE	0.001–1 µg/mL	97.4–100.3%	HPLC-FLD	[40]
Bupivacaine	-	-	40 min	LPME	0.1–10 µg/mL	88.9–93.3%	HPLC-UV	[41]
Buprenorphine	200 µL	700 µL	1 h20 min	Hydrolysis + Dilution + SLE	0.005–0.015 µg/mL	-	LC-MS/MS	[44]
Tramadol					0.05–0.15 µg/mL	-		
Buprenorphine	150 µL	465 µL	45 min	PPT	-	-	LC-MS/MS	[45]
Tramadol					-	-		
Tapentadol					-	-		

Abbreviations: Ref.: references; LPME–BE: liquid phase microextraction with back extraction; BS-DLLME: binary solvents dispersive liquid–liquid microextraction; DMSPE: dispersive micro-solid phase extraction; LPME: liquid phase microextraction; SLE: supported liquid extraction; PPT: protein precipitation; LC-MS: liquid chromatography–mass spectrometry; LC-MS/MS: liquid chromatography–tandem mass spectrometry; HPLC-UV: high-performance liquid chromatography-ultraviolet; HPLC-FLD: high-performance liquid chromatography coupled to fluorescence detection.

## Data Availability

The original contributions presented in this study are included in the article and Supplementary Material. Further inquiries can be directed to the corresponding author.

## Supplementary Materials

The following supporting information can be downloaded at: https://www.mdpi.com/article/10.3390/molecules31040605/s1, Figure S1: The distribution chart showing the concentration of different molecular species (microspecies or macrospecies) as a function of pH between 0 and 14; Figure S2: Influence of addition of NaCl and MeOH to the sample before SSµE; Figure S3: Chromatograms of three urine samples, a spiked urine sample at 210 nm (a), an unknown sample positive for articaine at 280 nm (b) and a blank urine sample at 210 nm (c); Table S1: Precision, Accuracy and Recovery.
