# Supplementary material for: Sorbent Strip Microextraction as a Practical Tool for Drug Screening: Application to Opioids and Local Anesthetics in Human Urine
Source: Molecules. 2026 Feb 9;31(4):605. doi: 10.3390/molecules31040605 (PMC12943546; doi:10.3390/molecules31040605)
Supplement: Supplementary file 1 [file molecules-31-00605-s001.zip › molecules-4116643-supplementary.pdf]

Supplementary Data

Buprenorphine

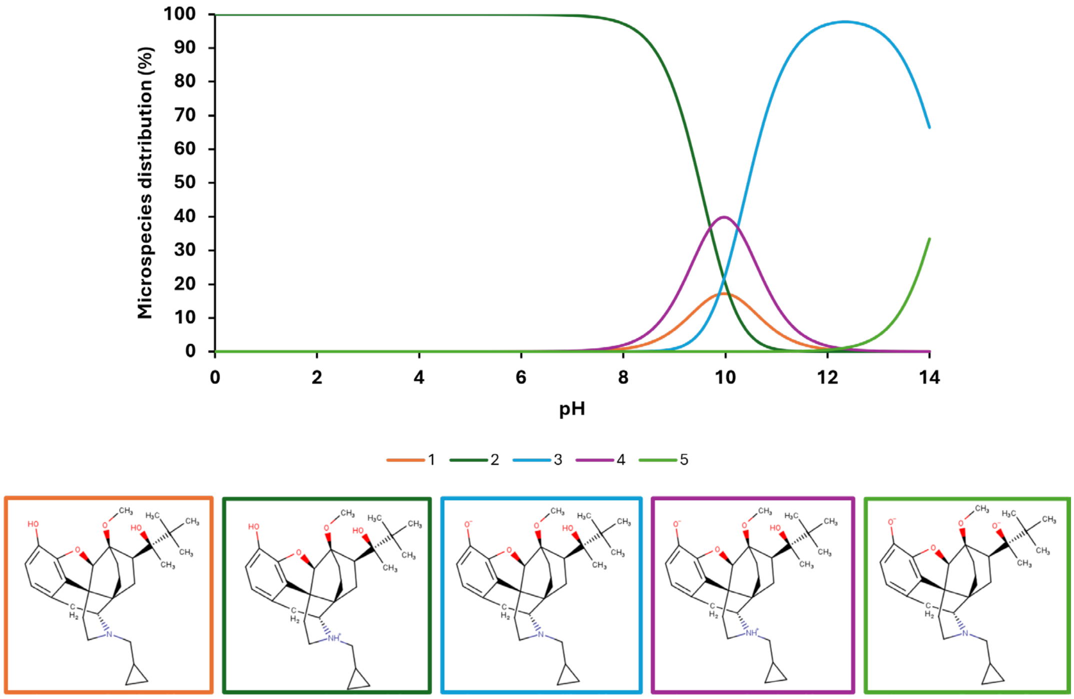

Tapentadol

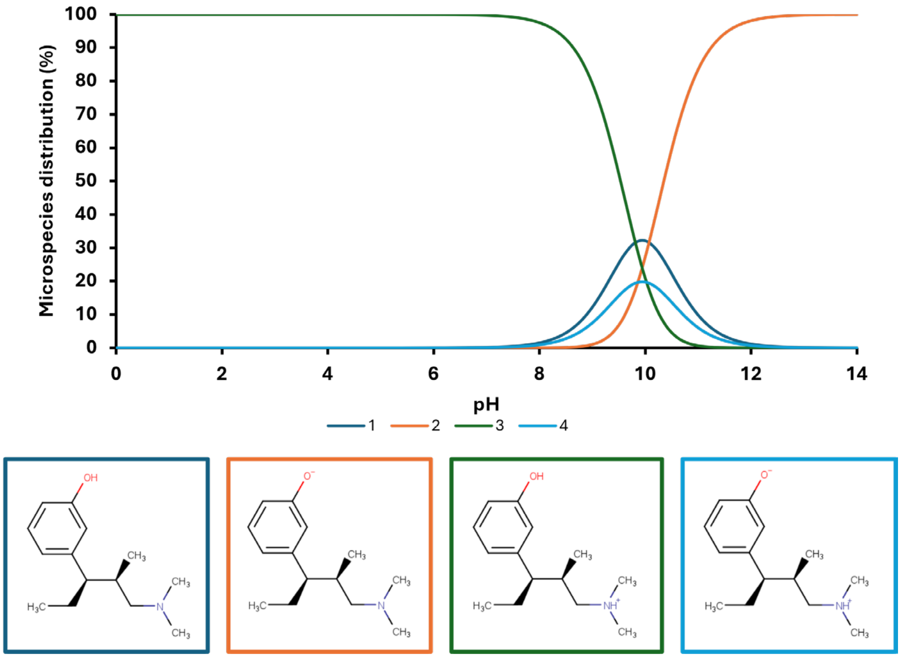

Tramadol

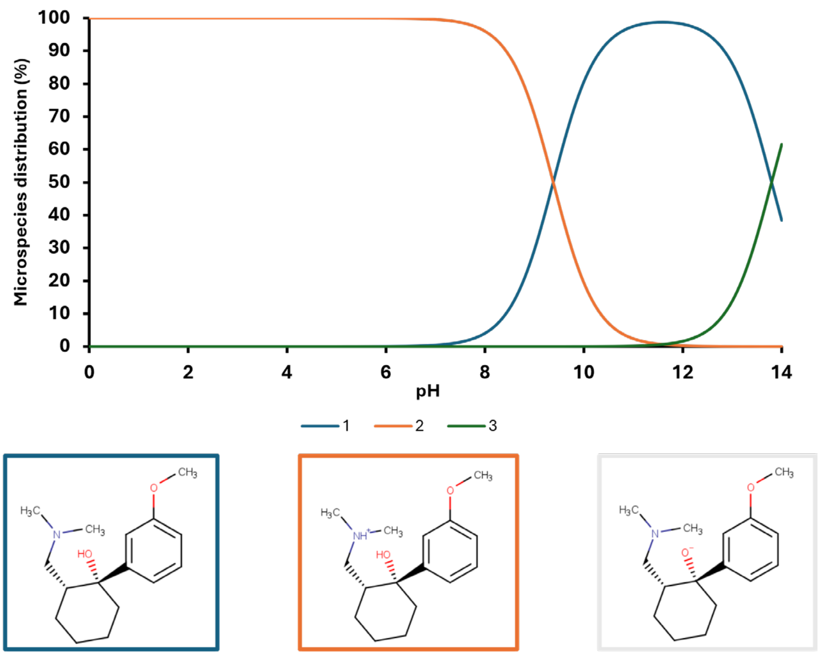

Articaine

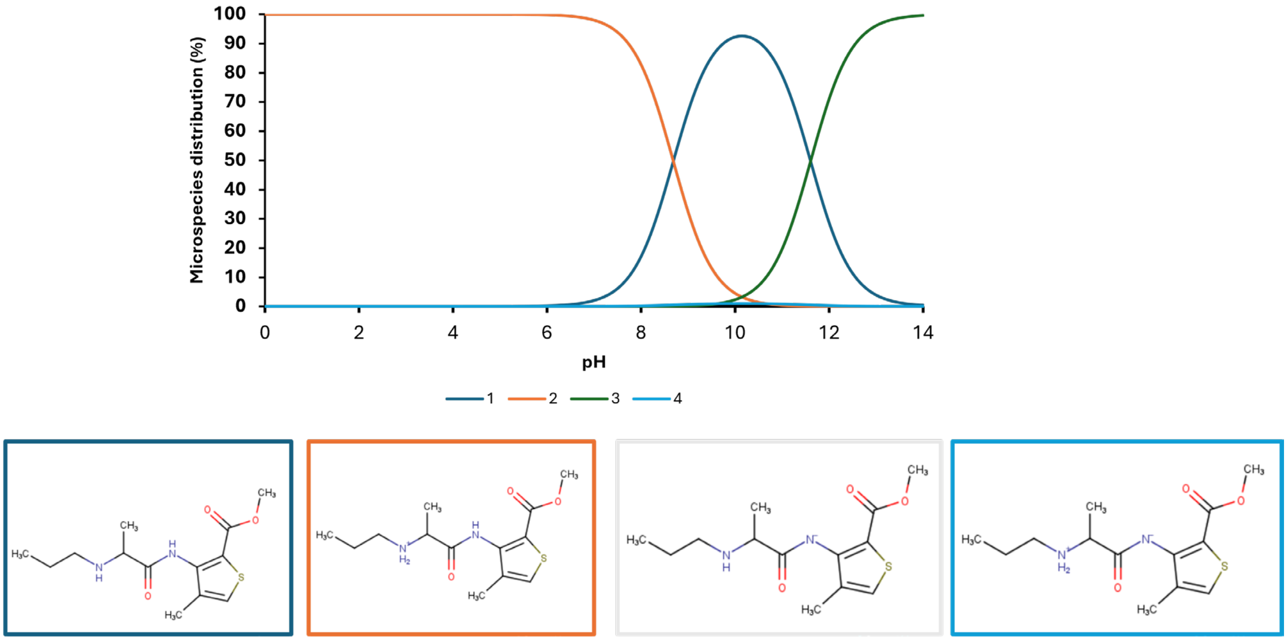

Bupivacaine

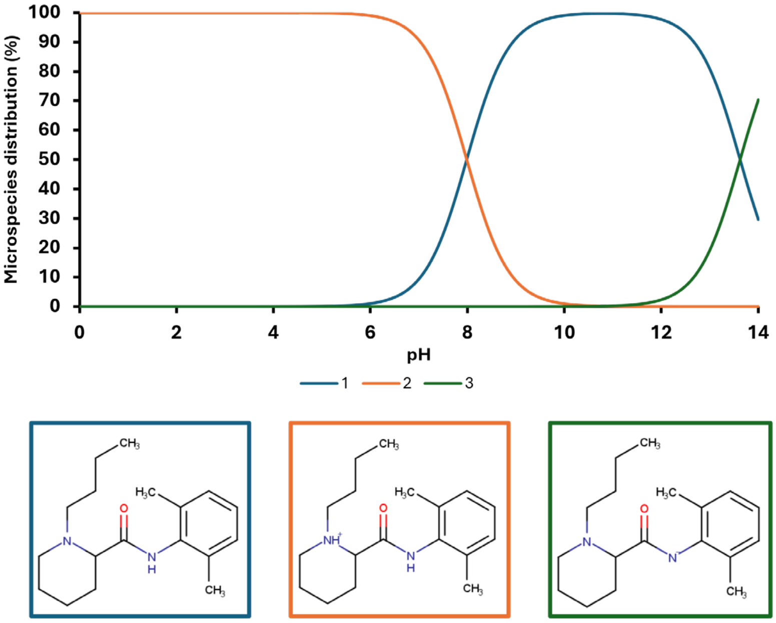

Figure S1. The distribution chart showing the concentration of different molecular species (microspecies or macrospecies) as a function of pH between 0 and 14. Marvin was used for drawing the chemical structures: Marvin 17.21.0, Chemaxon (https://www.chemaxon.com).

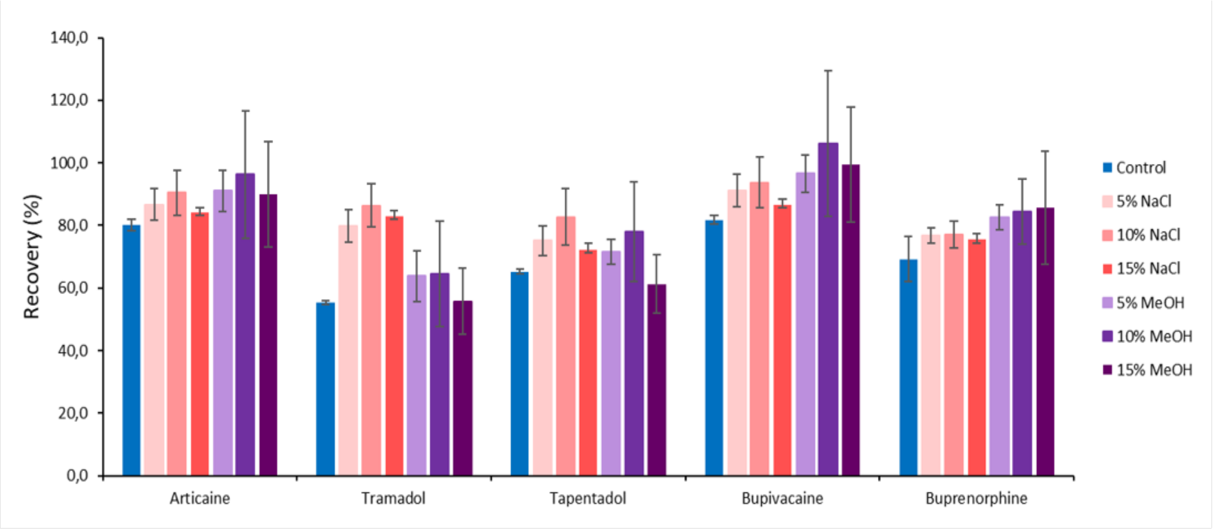

Figure S2. Influence of additon of NaCl and MeOH to the sample before SSμE.

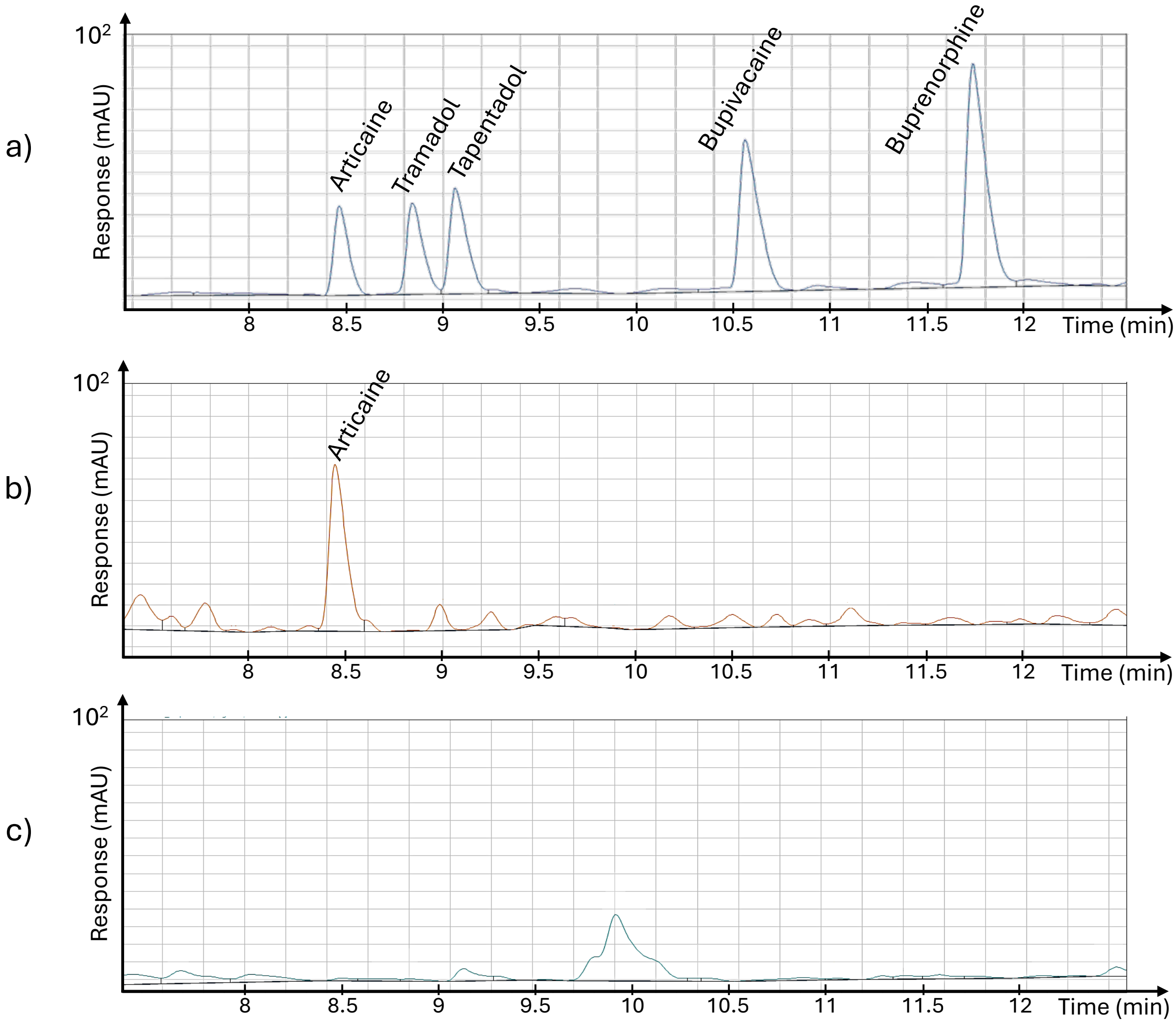

Figure S3. Chromatograms of three urine samples, a spiked urine sample at 210 nm (a), an unknown sample positive for articaine at 280 nm (b) and a blank urine sample at 210 nm (c).

Table S1. Precision, Accuracy and Recovery.

Compounds	Spiking Level (μg/mL)	Intra-day (n=6)		Inter-day (n=15)			
		Precision (%)	Accuracy (%)	Precision (%)	Accuracy (%)	Recovery (%)	RSD (%)
Articaine	1.5	1.6	2.0	2.2	1.3	86	2
	2.5	1.8	-1.8	3.4	-1.2	84	3
	4.5	4	0.3	4.6	0.2	82	5
Tramadol	15	3.9	3.6	12.7	5.7	103	9
	25	3.2	-3.2	9.1	-5.2	86	7
	45	4.4	0.6	9.6	1	78	8
Tapentadol	15	2	2.3	6.1	1.5	64	7
	25	1.6	-2.1	4	-1.3	73	5
	45	4	0.4	5.8	0.2	77	6
Bupivacaine	1.5	3.8	1.8	8.8	1.4	38	19
	2.5	4.4	-1.6	5.7	-1.2	57	9
	4.5	5.2	0.3	8.4	0.2	67	10
Buprenorphine	15	2	2.5	3.3	1.9	79	3
	25	2.7	-2.3	3.6	-1.7	80	3
	45	4.2	0.4	5.2	0.3	80	5
